# The Influence of the Dispersion Method on the Morphological, Curing, and Mechanical Properties of NR/SBR Reinforced with Nano-Calcium Carbonate

**DOI:** 10.3390/polym15132963

**Published:** 2023-07-06

**Authors:** Mehrnoosh Damircheli, AmirHossein MajidiRad

**Affiliations:** 1 Mechanical Engineering Department, Lafayette College, Easton, PA 18042, USA; 2 School of Engineering, University of North Florida, Jacksonville, FL 32224, USA; a.majidirad@unf.edu

**Keywords:** nanoparticles, nanocomposite, dispersion method, mechanical properties, material characterization, morphology

## Abstract

There are several reasons for the development of nanopolymer compounds, such as improving physical, mechanical, and chemical properties, increasing lifespan, reducing costs, and decreasing negative environmental impact. The compatibility of two rubbers and mineral nanofillers in nanocomposites is a challenge that needs to be studied, and the effect of nanofillers on morphological, physical, and mechanical properties should be investigated accordingly. In this study, calcium carbonate nanoparticles were added to a polymer compound that included natural rubber (NR), styrene-butadiene rubber (SBR), vulcanization accelerators, and other additives. For mixing nanoparticles in the polymer matrix, various methods were used, including the solvent method in toluene and W410 solvents and the surface modification of calcium carbonate nanoparticles with stearic acid. The effect of dispersion nanoparticles in nanocomposite specimens on morphology, curing characteristics, and mechanical properties was studied. The morphologies of specimens were determined by X-ray diffraction (XRD) analysis and field emission scanning electron microscopy (FESEM). The particle size of the nanocomposite was approximately 34 nm, and the interlayer spacing between crystal plates increased from 2.81 nm to 3.03 nm. These results indicate a uniform dispersion of nanoparticles, specifically with an optimum content of 3.52%, in the compounds prepared through all mixing methods, with no agglomeration observed in the nanocomposites. The results of the nanocomposites’ curing characterization demonstrate that with the addition of nanoparticles, a strong bond is created in the polymer chains, and curing properties are improved. Among the dispersion methods, the highest percentage improvement in curing properties is observed with the solvent method W410. To evaluate the effect of the addition of calcium carbonate nanoparticles and the dispersion method on improving mechanical properties, tensile, tear, hardness, and rebound resilience tests were performed. In tensile tests, the surface modification method showed the highest enhancement in ultimate stress (80%), followed by the W410 method (64%) and toluene method (63.7%). Tear strength improvements were highest in the W410-solvent sample (80%), followed by the surface modification method (57%) and the solvent-toluene method (50%). The W410 method resulted in the hardest samples, while the surface-modified samples had the lowest hardness. The addition of CaCO3 nanofillers reduced rebound resilience, with the W410 method experiencing the largest reduction (10.64%), followed by the toluene method (6.38%), and with the surface-modified samples showing the lowest reduction (4.25%). The results show that in the W410 solvent method, the nanocomposite is more elastic than for other methods. Additionally, for most of the mechanical properties, the W410 method results in the most growth in improvement.

## 1. Introduction

Natural rubber (NR) and styrene-butadiene rubber (SBR) are widely recognized as versatile elastomers, owing to their unique physical and mechanical properties, which make them suitable for a diverse range of industrial applications such as automotive tires, sealing materials, and dairy rubber items [1,2,3]. While carbon black is the most commonly used filler for reinforcing NR and SBR, nanosized fillers have shown the potential to enhance the mechanical properties of these elastomers due to fillers’ high surface area and unique physical properties. Researchers have thus explored the use of different types of nanofillers, including nanosilica, ultra-fine calcium carbonate, cellulose whiskers, clay aerogel, diatomaceous earth, and reduced graphene oxide, to modify NR vulcanizates in order to achieve improved thermostability and mechanical properties [4,5,6,7,8,9]. However, the extent of the improvement achieved depends on various factors, such as the type of nanofiller, particle size, weight ratio, method of dispersion, adsorption characteristics, and surface properties, all of which play a critical role in influencing the interaction between the filler and the matrix [10,11,12]. The use of polymer nanocomposites in industries is gaining significant attention due to the nanocomposites’ unique physical properties, including improved mechanical, chemical, and thermal properties. The performance enhancement of the elastomers can be attributed to the synergistic effects of the different types of fillers, which optimize intercomponent interactions and improve the thermomechanical properties of the material. The effectiveness of the filler/matrix interface is critical to achieving effective stress transfer and further enhancing the thermomechanical properties of the composites [13,14].

The reinforcement of polymers with nanoscale particles has emerged as a promising strategy for producing high-performance materials. Among the mineral fillers available, calcium carbonate (CaCO_3_) has attracted attention owing to its low cost, abundance, and whiteness [15]. However, traditional CaCO_3_ particles suffer from large particle–particle interactions, leading to inhomogeneous distribution, processing difficulties, poor appearance, and inferior properties in rubber composites. To overcome these limitations, researchers have investigated the use of nanoscale CaCO_3_ particles. Incorporation of nano-CaCO_3_ into various polymer matrices has been shown to significantly enhance their mechanical and physical properties. For example, in acrylonitrile-butadiene-styrene, nano-CaCO_3_ improved modulus and impact strength [16], while in epoxy resin, it enhanced compressive strength, elastic modulus, and elongation [17]. Similarly, in polypropylene, nano-CaCO_3_ increased fractural toughness [18,19], and in high-density polyethylene, it improved tensile strength [20]. These improvements were also observed in polyvinylchloride [21], poly(propylene carbonate) [22], natural rubber [5,23,24], SBR [25], ethylene-propylene-diene rubber [26], acrylonitrile-butadiene rubber [27], and butadiene rubber [28]. In the surveyed literature, researchers have reported particle size ranges of nano CaCO_3_ typically ranging from 30–60 nm, demonstrating its versatile applications as a filler material. The findings of these studies underscore the vast potential of nano-CaCO_3_ as a filler material for developing high-performance rubber composites.

The dispersion method employed is crucial in the production of nanocomposites with superior properties. CaCO_3_ nanoparticles have a small particle size, large specific surface area, high surface energy, and hydrophilic and oleophobic properties, which can result in agglomeration and limit their use in various materials. The hydrophilic nature of CaCO_3_ nanoparticles, in particular, poses challenges in achieving uniform and compatible dispersion in hydrophobic polymers. Researchers have explored several methods, including ball milling, ultrasonic treatment, and surface modification, to improve the dispersion of nano-CaCO_3_ in natural rubber and polymer matrices. Ball milling and ultrasonic treatment are effective techniques to decrease particle size and prevent the formation of agglomerates of fillers [29]. Combining ultrasonic and ball milling methods can further reduce particle size and prevent agglomeration, leading to improved mechanical and thermal properties of nanocomposites [30]. However, surface modification techniques have gained significant attention in recent years due to their ability to enhance compatibility between the filler and polymer matrices. Various surface modification techniques, such as silane coupling, surface grafting, and plasma treatment, have been investigated to improve the dispersion of nano-CaCO_3_ in natural rubber and polymer matrices. The use of modified CaCO_3_ has also been explored to improve dispersion in natural rubber films [31,32]. The weight percentage of nanofillers is another critical factor that affects the mechanical properties of nanocomposites. Various studies have investigated the impact of filler content on the properties of polymer-based nanocomposites. It has been shown that the addition of a small amount of nano-CaCO_3_ (less than 5 wt%) can significantly improve the mechanical properties of the resulting nanocomposites [31,33]. Therefore, careful optimization of the filler content, dispersion methods, and surface modification techniques is essential to achieve the desired mechanical properties in nanocomposites filled with nano-CaCO_3_.

The development of nanocomposites is a promising approach to improve the mechanical, physical, and environmental properties of polymers. In this study, we investigate the compatibility of a polymer compound consisting of natural rubber (NR), styrene-butadiene rubber (SBR), stearic acid (ST.AC), paraffin wax (Pwax), aromatic oil (AR OIL), carbon black (CB N500), zinc oxide (ZnO), vulcanization accelerator cyclohexyl benzothiazole sulfenamide (CBS), sulfur, p-Phenylenediamine (6PPD), and 2-dihydroquinoline (TMQ), with calcium carbonate nanoparticles (nano-CaCO_3_), as a nanofiller to produce nanocomposites with improved properties. The dispersion of nanoparticles in the polymer matrix was achieved using various methods, including solvent methods in toluene and W410 solvents (a hydrocarbon-based solvent composed of paraffinic compounds with a carbon chain length ranging from C5 to C8) and surface modification of nanoparticles with stearic acid. The morphological, curing, and mechanical properties of the nanocomposites were studied to determine the best dispersion method. This investigation focuses on novel techniques to achieve uniform dispersion of nanoparticles in the polymer matrix, contributing to the advancement of nanocomposite research. X-ray diffraction and scanning electron microscopy images revealed uniform dispersion of nanoparticles in all mixing methods, and the curing properties of the nanocomposites were improved with the addition of nanoparticles. This finding demonstrates the positive impact of nanofillers on the curing process and emphasizes their potential in enhancing the overall performance of nanocomposites. In our preliminary study, it was found that lower weight percentages, such as 2%, did not significantly alter the properties, while higher percentages resulted in agglomeration. Therefore, the optimal content of 3.52% was determined for achieving desirable effects without compromising dispersion quality.

This critical finding provides valuable insights into the appropriate nanofiller content necessary to maintain the integrity and performance of the nanocomposites. Among the dispersion methods, the W410 solvent method demonstrated the most significant improvement in the mechanical properties of the nanocomposites. This notable outcome highlights the superiority of the W410 solvent method in enhancing the mechanical performance of the nanocomposites. This investigation of the effect of various dispersion methods on the properties of NR/SBR nanocomposites with calcium carbonate nanoparticles has the potential to lead to the development of new materials with improved properties. By combining innovative dispersion techniques with the optimal nanofiller content, our research offers new perspectives in the field of nanocomposite development and paves the way for future advancements.

## 2. Materials and Methods

### 2.1. Materials

Consumable materials include natural rubber with styrene-butadiene (NR/SBR), stearic acid (ST.AC), calcium carbonate nanoparticles (CaCO_3_), paraffin wax (Pwax), aromatic oil (AR OIL), carbon black (CB N500), zinc oxide (ZnO), vulcanization accelerator cyclohexyl benzothiazole sulfenamide (CBS), sulfur, p-phenylenediamine (6PPD), and 2-dihydroquinoline (TMQ).

Table 1 shows the amounts of the materials used in terms of weight, PHR (parts per hundred rubber), and weight percentage for the preparation of nanocomposites. The physical properties of calcium carbonate nanoparticles are given in Table 2.

### 2.2. Nanocomposite Preparation

In this research, different methods for the dispersion of calcium carbonate nanoparticles in the matrix of natural rubber and butadiene rubber were investigated. Several different methods were used to ensure proper nanoparticle dispersion.

One of the methods used for uniform dispersion of nanoparticles in composites is the solvent method. In this method, two solvents, toluene and W410, were used. SBR and NR were cut and poured into a container to mix with solvents. The amount of solvent added to the samples is shown in Table 3.

**Figure 1:**
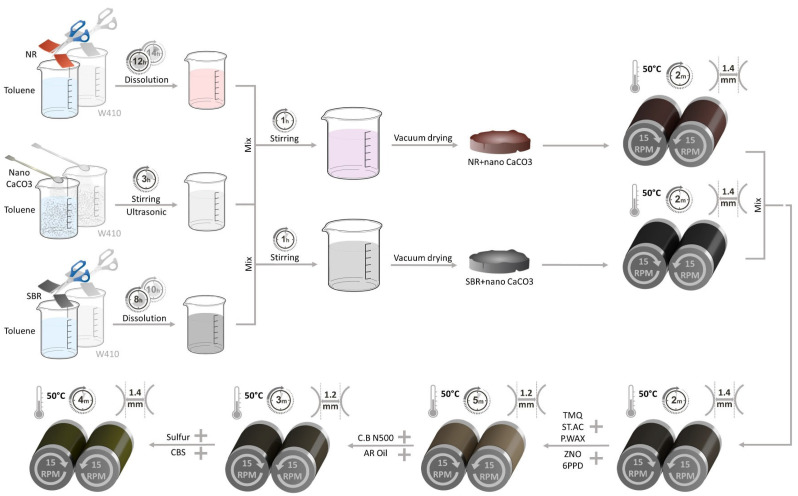
Flow chart of nano-composites prepared by solvent methods.

The gelling process continued over time; the volume of the gelling mode in both rubbers (NR and SBR) increased, and after 2 h, about 30% of the samples had become gels. Preliminary results indicate that both solvents had a greater effect on SBR than NR. In addition, gelling in toluene was more advanced than in W410. In other words, toluene had more penetration on the rubbers than W410, and the highest volume of gelling was related to styrene-butadiene rubber. As a result, by continuing this process, the gelled state of SBR was obtained with a suitable emulsion through the solvent method; then, NR was completely gelled in the solvents. A powder of calcium carbonate nanoparticles was added to 200 mL of solvent and spread by an ultrasonic stirrer for up to 2 h at 1000 rpm. A stable and uniform solution was obtained and finally added to the gel-mixed NR and SBR in the solvents, and a mechanical stirrer helped to create a complete uniform distribution, which was then dried in a vacuum oven. The outcome material was a combination of NR and SBR with calcium carbonate nanoparticles. All the rubber nanocomposites were prepared with a two-roll mill. The process parameters, including but not limited to the temperature, the distance between the rollers, and the ingredient that should be added at each step, are shown in Figure 1.

To prepare the surface-modified specimens, first, a 250-mL beaker containing aromatic oil and paraffin wax was heated to 60 °C on a hot plate in a water bath while being mixed with an ultrasound stirrer at a rotation speed of 250 rpm. Then, stearic acid was added to the mixture. Finally, after ensuring that the components were properly mixed, nano-CaCO_3_ was gradually added to the mixture. By increasing the blade rotation speed up to 1200 rpm for 20 min at 60 °C, complete mixing of the nanoparticle calcium carbonate was ensured at this step. Typically, after this homogenized mixture is prepared, carbon black would be needed to form a compound. For our experiment, after rolling the NR and SBR rubbers according to the flow chart shown in Figure 1, we added other ingredients; instead of adding carbon black, we used a new mixture of aromatic oil, paraffin wax, stearic acid, nano-CaCO_3_, and CB N500. We refer to the prepared nanocomposite as the surface-modified sample, since the surface of the nanoparticles is modified by stearic acid.

### 2.3. Characterization

#### 2.3.1. X-ray Diffraction (XRD) Analysis

To analyze the crystal structure of CaCO_3_ nanoparticles and other non-amorphous crystalline compounds such as zinc oxide in this rubber matrix, XRD analysis was used [34]. According to standard BS EN 13925-10, the X-ray diffraction study was carried out using an X’Pert-MPD PANalytical X-ray diffractometer (X’PertMPD, PANalytical, The Netherlands) in the range of diffraction angle 2θ = 1–100° with a scanning speed of 0.02 deg/sec. Copper was used as a target material for single-crystal diffraction, with CuKɑ radiation (λ = 0.154 nm). This analysis was performed using a CuKɑ filter at 22 °C with 44% humidity. An accelerator voltage of 40 kV and a beam current of 30 mA were used. The *d*-value (distance of covalent or electrostatic bond between two atoms) of calcium carbonate (CaCO_3_) nanoparticles was calculated using Bragg’s law (λ = 2dsinθ).

#### 2.3.2. Field Emission Scanning Electron Microscopy (FESEM)

Morphology analysis of non-nanocompounds and nanocomposites was performed with field emission scanning electron microscopy (FESEM-TESCAN-Model MIRA3, voltage 15 kV). This analysis was used to study the morphology of the fracture surface of the polymer composite samples under tensile testing. The fracture surfaces were coated with platinum before imaging by the device.

#### 2.3.3. Cure Characteristics

To investigate the curing properties of the mixtures, following the ASTM D2084 standard, an ODR rheometer (Zwick 4308 model) at 160 °C and with an oscillation angle of 1° was used to measure the properties. The test duration was 20 min, and the torque range was selected in the range of 0−100 lbf.in.

In this test, a sample of the vulcanizable rubber compound was placed in the cavity of the molds and sealed under positive pressure. The conical oscillating disc of the rheometer was surrounded by a sample that had reached the curing temperature. The disk then oscillated sinusoidally at angles of 1° and a frequency 1.7 Hertz. While the Moni viscometer’s rotor rotates at a constant speed and direction, the difference in this motion causes a shear strain in the sample. Torque and temperature changes over time were recorded. The torque required for disk oscillation depends on the shear modulus of the compound. The shear modulus increases as the bonds between the polymer chains increase and strengthen during the vulcanization process.

The parameters scorch time 5 $\left(t_{S5}\right)$ and scorch time 10 $\left(t_{S10}\right)$; cure times 90, 95, and 100% $\left(t_{90},\;t_{95},\;t_{100}\right)$; minimum torque $\left(M_{L}\right)$, maximum torque $\left(M_{H}\right)$, and the difference between the maximum and minimum torques $\left(M_{H}-M_{L}\right)$; peak cure rate $\left(V_{\max}\right)$ and cure rate index; and reinforcement efficiency of the filler $\left(\alpha_{f}\right)$ are the extracted results of this test.

#### 2.3.4. Mechanical Properties—Tensile Test

**Figure 2:**
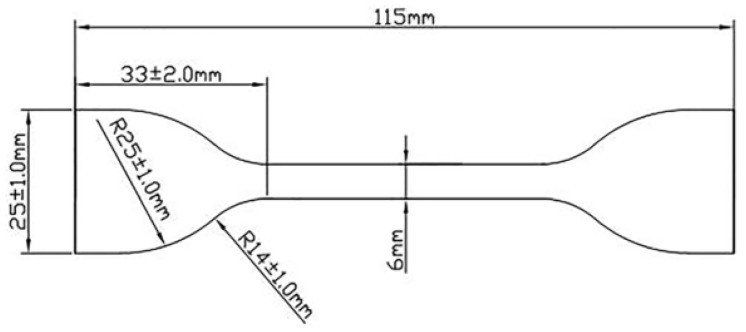
Dimensions of the tensile test sample according to ASTM D 412 standard.

Mechanical properties related to tensile tests, including ultimate stress, fracture strain or elongation at break, and Young’s modulus for the above nanocomposites were measured by testing dump-shaped samples punched from the final cured compound sheet. This test was performed by a Hiwa tensile testing machine according to the ASTM-D412 standard at room temperature. In this test, the jaws of the machine moved away from each other at a constant speed of 500 mm/min to stretch the specimen. To ensure the repeatability of the test, five measurements were recorded. Other sample parameters such as thickness and length related to the initial gage length in the narrow part of the test piece are considered 1.3 to 3.3 (2 mm) and 25 ± 0.25 mm, respectively. Figure 2 shows a drawing of the tensile test sample and its dimensions.

#### 2.3.5. Mechanical Properties-Tear Test

**Figure 3:**
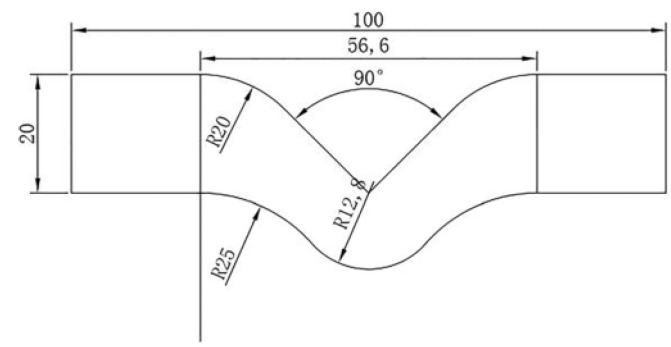
Dimensions of the tear test sample according to ASTM D 624 standard.

This test was performed by a Hiwa tensile testing machine with a jaw speed of 500 mm/min according to the ASTM D 624 standard. The same machine as used in the tensile test was used to measure the specimen’s tear strength, in both the longitudinal and transverse directions, by changing the jaws. This test is important since it determines how long a tire can withstand a tear when a sharp object hits it, which is critical for all kinds of tires. Samples for the tear test were punched out from molded sheets, as shown in Figure 3.

#### 2.3.6. Mechanical Properties—Hardness Test

The hardness of a material is defined in terms of its resistance to depression by a harder object. To measure the hardness of soft rubbers, a Shore A hardness tester (12.5 N load) was used, and for hard rubbers, a Shore D tester (50 N load) was used. This Shore A hardness test was performed by a Zwick machine according to ASTM D2240 or DIN53505 standards. The measured hardness can be considered a measure of the elastic modulus in small deformations.

#### 2.3.7. Mechanical Properties—Resilience Test

The dynamic behavior of materials, the degree of elasticity, and the material behavior after fatigue and aging tests are among the factors that affect the rebound resilience of rubbers. In other words, this resilience test determines the amount of rebounding, flexibility, and change in elastic behavior of rubbers after exposure to aging. This test was performed according to the requirements of the ASTM D1054 standard by the KARL FRANK Model 23567 machine, which measures the amount of energy loss during a short impact deformation. For this purpose, a pendulum hammer with a diameter of 15 mm was dropped from a certain height and struck the sample with a speed of 2 m/s, carrying 0.5 J of energy. The height that the hammer rises after impact is the criterion of evaluation, for which two samples with dimensions of 25 ± 0.5 × 25 ± 0.5 × 50 ± 1 mm were required. The test temperature was 23 ± 2 °C. According to ASTM D1054, six tests are performed and the average of the last three tests must be reported (for each specimen, the pendulum hits three different locations).

## 3. Results and Discussion

### 3.1. X-ray Diffraction

After X-ray diffraction analysis, the presence of two crystalline structures in the nanocompound was identified. In the non-nanocompound, this structure is related to zinc oxide, and in the nanocomposite (W410 sample), structures are related to zinc oxide and calcium carbonate nanoparticles. As mentioned above, the interlayer space between the crystal plates of CaCO_3_ nanoparticles is calculated by Bragg’s equation:

(1) λ=2d.sin(θ)

In Equation (1), λ, d, and θ denote the X-ray wavelength of Copper K-α, the interplanar distance located in adjacent Bragg’s planes in the angstrom range, and the angle between the incident ray and the relevant crystal planes, respectively. Based on the diffraction analysis, the crystalline phase of calcite (Ca6.00C6.00O18.00), which has a mainly hexagonal crystal structure and is in accordance with calcium carbonate nanoparticle properties, has been identified (Table 2).

**Figure 4:**
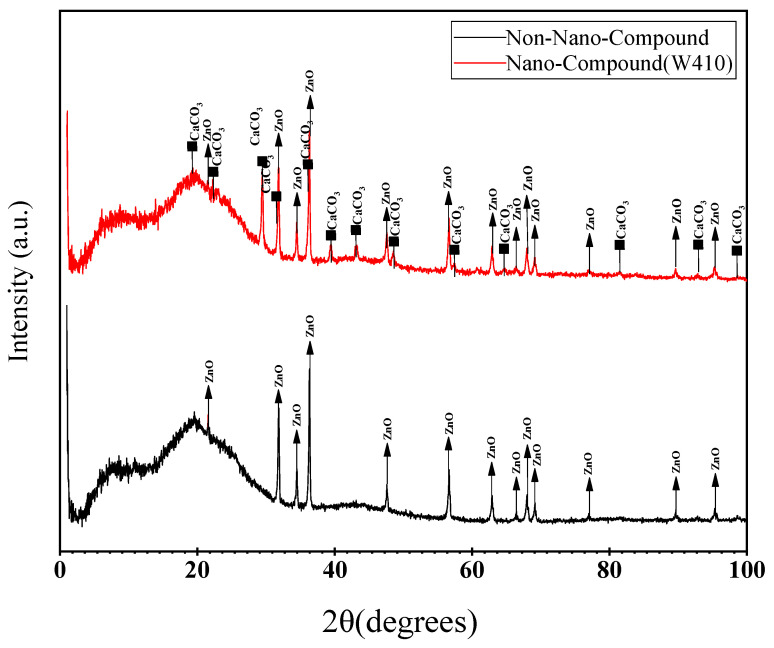
X-ray diffraction patterns of control compound and nanocomposites (W410 sample).

The diffraction diagrams of the nanocomposite and non-nanocompound were determined and are shown in Figure 4. The XRD pattern of ZnO was analyzed by Xpert software, and the characteristic peaks appeared at different 2*θ* values (31.8, 34.4, 36.3, 47.53, 56.61, 62.87, 66.34, 67.98, 69.1, 72.63, 77.04, 81.39, 89.55, 92.57, and 95.34 degrees), which is in good agreement with the electron diffraction results of ZnO nanoparticles according to the Joint Committee on Powder Diffraction Standard (JCPDS) card No. 89-1397 [35]. ZnO nanocrystals have three similar characteristic peaks at 2*θ* values (31.8, 34.4, and 36.3 degrees), which correspond to the hexagonal lattices (1 0 0), (0 0 2), and (1 0 1), respectively.

The XRD pattern of the nanocomposite compound has peaks at different 2*θ* values (23, 29.4, 31.8, 36.2, 39.4, 43.1, 47.5, 48.5, 49, 56.6, 57.4, 66, 77, 81, and 83 degrees). It was concluded that all of the peaks obtained in the XRD pattern of nano-CaCO_3_ perfectly match with the standard calcite pattern and JCPDS card No. (88-1807). It is important to note that the XRD pattern for the nanocomposite sample is a combination of the peaks associated with both ZnO and CaCO_3_, and due to the proximity of some characteristic peaks at equal angles, the overlap of some peaks is observed. The crystalline phases of both the calcium carbonate and zinc oxide are well detectable in the nano-reinforced composite. The change in the absorption peak of the active groups that have the potential to interact with the nanoparticles indicates the presence of nanoparticles [36].

In addition, according to the Debye Scherrer equation, the size of the crystalline nanoparticles at the maximum diffraction intensity )2*θ* = 29.41 degrees) is about 34 nm, which corresponds to the structural characteristics of the nanoparticles used here (Table 2) [37].

(2) $$D=\frac{K\lambda }{\beta \cos\theta }$$

In the Debye Scherrer equation, *θ*, D, λ, *β*, and K are particular diffraction angles in radians (not degrees) and correspond to half of 2*θ* (typically plotted in an XRD pattern), the crystalline domain size in nanometers; the X-ray wavelength in nanometers (which is constant and depends on the type of X-rays used; in this case, it was the X-ray wavelength of Copper K-α and is 0.154 nm); the width of the peak at half of its height, as radians; and the Scherrer constant (which is typically considered to be 0.89) [38].

The broad peak in the XRD pattern clearly shows that small nanocrystals exist in the samples. There is no evidence of bulk materials or impurity. Peaks appear at different 2*θ*, as shown in Figure 4. The sharp diffraction peaks show the good crystallinity of the CaCO_3_ nanoparticles and ZnO.

In the spectrogram of the non-nano-compound, an intense characteristic reflection at 2*θ* = 31.8°, corresponding to a basal spacing of 2.81 nm, is observed. By adding calcium carbonate nanoparticles to the polymer compound, an increase in the intensity of X-ray diffraction at the first peak in the compound containing CaCO_3_ nanoparticles compared to the control compound can be observed. Meanwhile, with the addition of nanoparticles, the interlayer space between the crystal plates increases from 2.81 nm to 3.03 nm. This result indicates the breaking of agglomeration and good dispersion of CaCO_3_ nanoparticles. Effective force pushing the rubber chains into the interlayer spaces and causing an increase in interplanar distance leads to proper dispersion of the calcium carbonate nanoparticles. CaCO_3_ particles agglomerate easily due to their small particle size, high specific surface area, and surface free energy [39]. In addition, the surface of CaCO_3_ particles has hydroxyl groups that show polarity and have hydrophilic properties that hinder uniform dispersion of nanoparticles in the organic matrix, but the particle size obtained in the nanocomposite by XRD and the sharp diffraction peaks are evidence of good dispersion.

### 3.2. Field Emission Scanning Electron Microscopy (FESEM)

**Figure 5:**
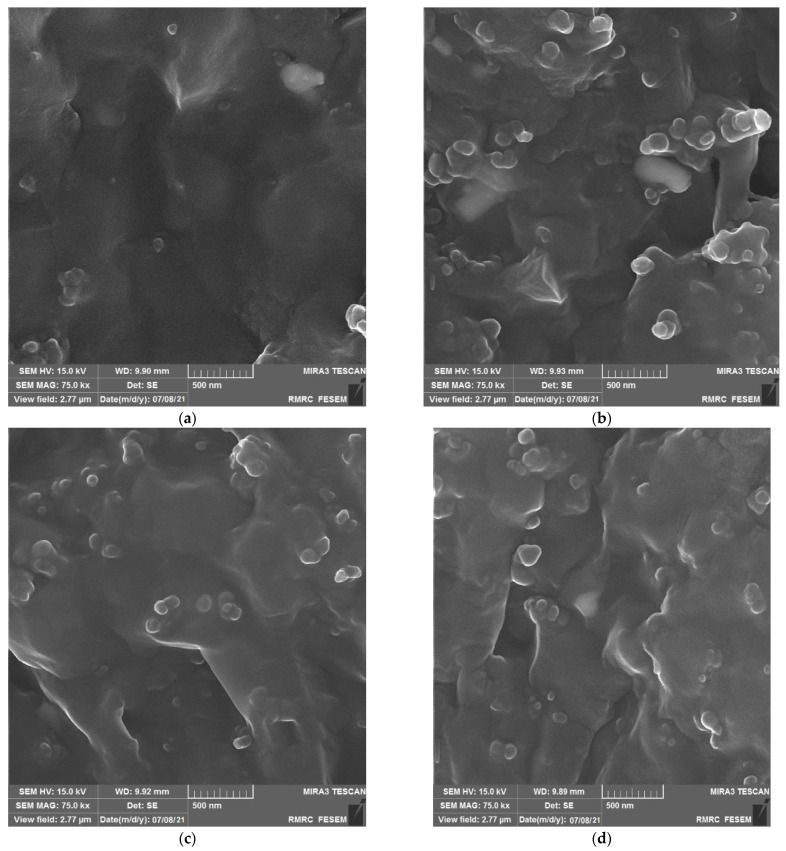
FESEM images: (**a**–**d**) represent the FESEM images of the control compound, solvent-W410, surface-modified, and solvent-toluene nanocomposites.

The scanning electron microscope images of the composite without filler and the samples containing nanoparticles at a 75,000 magnification scale are shown in Figure 5. As these images show, in all dispersed methods (surface-modified, solvent-W410, and solvent-toluene), the particle sizes of nano-CaCO_3_ are within the range of 10 to 60 nm, which is in good agreement with the XRD results and the specifications of calcium carbonate nanoparticles listed in Table 2. Another important point is the very good dispersion of particles in the samples prepared by the surface-modified method and solvent methods, which can lead to improved physical and mechanical properties in these samples. This will be discussed in detail below. The figures show no sign of agglomeration of calcium carbonate nanoparticles, and the size of the nanoparticles is at the nanoscale, indicating good dispersion of nanoparticles across all dispersion methods.

### 3.3. Cure Characteristics

**Figure 6:**
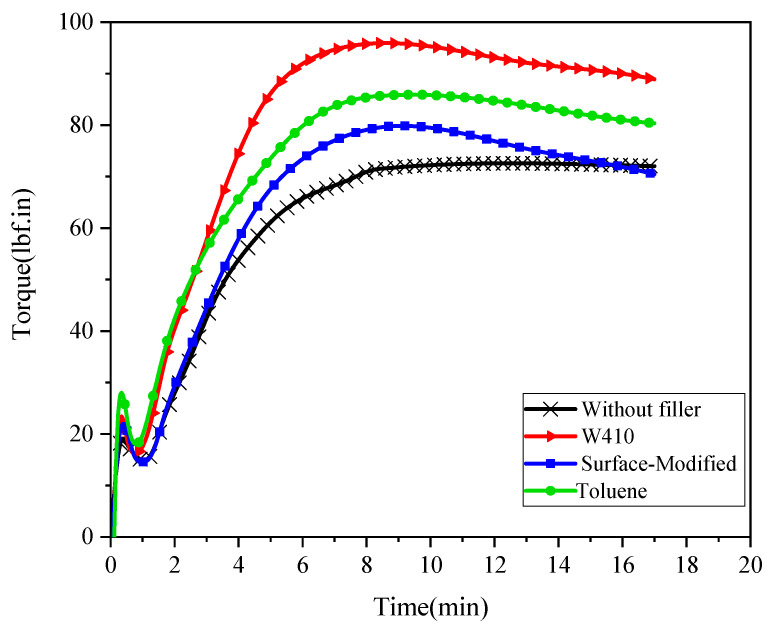
Cure curves (torque vs. time) for without-filler compound and nanocomposites.

Table 4 lists the curing properties associated with various methods of dispersing the reinforcing nanoparticles in the polymer matrix and compound without filler. All curing parameters are extracted from the torque-time diagram in Figure 6.

The maximum torque $\left(M_{H}\right)$ is a measure of the completely cured composite’s stiffness and shear modulus at the curing temperature. The strong interaction between the polymer chains and the filler increases the maximum torque. The torque difference $\left(M_{H}-M_{L}\right)$ is also a criterion based on the difference in stiffness or shear modulus between the cured and uncured samples, which can be considered a measure of the chemical cross-link between the matrix and the filler. Minimum torque $\left(M_{L}\right)$ is a parameter used to evaluate the degree of physical entanglement of an uncured sample’s polymer chains.

As the data in Figure 6 and Table 4 show, the samples with calcium carbonate nanoparticles (3.52 wt%) in all dispersion methods demonstrate a significant increase in maximum torque $\left(M_{H}\right)$ and in the difference between the maximum and minimum torque $\left(M_{H}-M_{L}\right)$ compared to the mixture without reinforcing nanoparticles. The reason for this increase is the restriction of the compound’s chain mobility due to the formation of strong interactions between the added calcium carbonate nanoparticles and the polymer matrix. Previous research has reported that the addition of calcium carbonate nanoparticles to NR causes increased maximum torque and a larger difference between the maximum and minimum torque [5,24]. Further investigation shows that among the dispersion methods of reinforcing nanoparticles in the compound, the percentage of increase in the above parameters is strongly dependent on the pre-dispersion methods of composite components. Thus, the highest percentage increase of $\left(M_{H}\right)$ and $\left(M_{H}-M_{L}\right)$ parameters in nanocomposites is seen with the use of W410, followed by toluene, and the lowest percentage increase is seen with the surface-modified method. In addition, by adding calcium carbonate nanoparticles in all methods, the minimum torque also increases compared to the non-reinforcing nanoparticle compound due to a strong interaction between the nanoparticles and the matrix polymer and a restriction in chain motions.

By analyzing other results of the rheometer test, depicted in Figure 6 and Table 4, scorch times, cure times, peak cure rate (maximum slope to the cure curve), and cure rate index are derived. The addition of nanoparticles in all dispersion methods reduces scorch times and cure times. This reduction is attributed to an increase in the speed of the curing process. According to the results of this test, the best performance is associated with the highest reduction in scorch and cure times. Therefore, the solvent-W410 method produces the fastest curing process. Meanwhile, peak cure rate and cure rate index are also increased by adding nanoparticles across all methods, with the solvent-W410 method showing the largest increase. Looking at all parameters of the rheometer test and the curing properties, it can be concluded that the addition of calcium carbonate nanoparticles to the compound significantly improves curing properties.

(3) $$\mathrm{CRI}=\frac{100}{\mathrm{cure}\;\mathrm{time}-\mathrm{schorch}\;\mathrm{time}}=\frac{100}{t_{95}-t_{S10}}$$

In the above equation, CRI, $t_{95}$, and $t_{S10}$ are cure rate index, cure time 95%, and scorch time 10, respectively.

Another important parameter related to the reinforcing effect of nanoparticles is the reinforcement efficiency of the filler. As mentioned above, the difference between minimum and maximum torque is a measure of the variation in stiffness or shear modulus between the cured and uncured samples, which increases with the addition of nanoparticles. This is also shown by the results presented in Table 4. Equation (4) explains the relationship between reinforcement efficiency of the filler and torque increase, as follows:

(4) $$\alpha_{f}=\frac{\frac{\Delta M_{c.w.f}}{\Delta M_{w.o.f}}-1}{wt\%}$$

In the above equation, $\Delta M_{c.w.f}$, $\Delta M_{w.o.f}$, and wt% are the difference between the maximum and minimum torques of compounds with filler (c.w.f), the difference between the maximum and minimum torques of without-filler compounds (w.o.f), and the weight percentage of nanoparticles, respectively. In this study,wt% is 3.52%. This parameter illustrates the activity of nanoparticles in the matrix. The large value of this coefficient indicates a strong interaction and bond between the nanoparticles and the compound.

**Figure 7:**
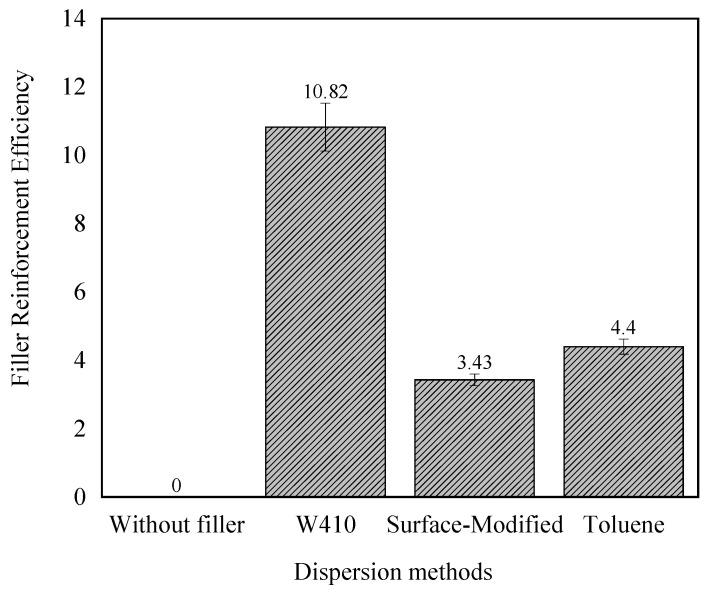
The reinforcement efficiency of the filler for the control compound and the solvent-410, surface-modified, and solvent-toluene nanocomposites.

Figure 7 shows the reinforcement efficiency of the filler for several dispersion methods. The results show that this coefficient is highest in the W410-solvent method. These results correspond to the curing properties listed in Table 4. The results are in good agreement with previous studies, where it has been found that the rheological properties of epoxy composites with graphene or graphite nanoplatelets increase exponentially with filler loading [40]. Other researchers have found that bound rubber between natural rubber and nano- and micro-calcium carbonate exponentially improves with increasing amounts of calcium carbonate [23]. Finally, it is important to note that with the addition of nanoparticles, a strong bond occurs in the polymer chains, and among the dispersion methods, the strongest bond is obtained in the 410-solvent method.

### 3.4. Mechanical Properties—Tensile Test

**Figure 8:**
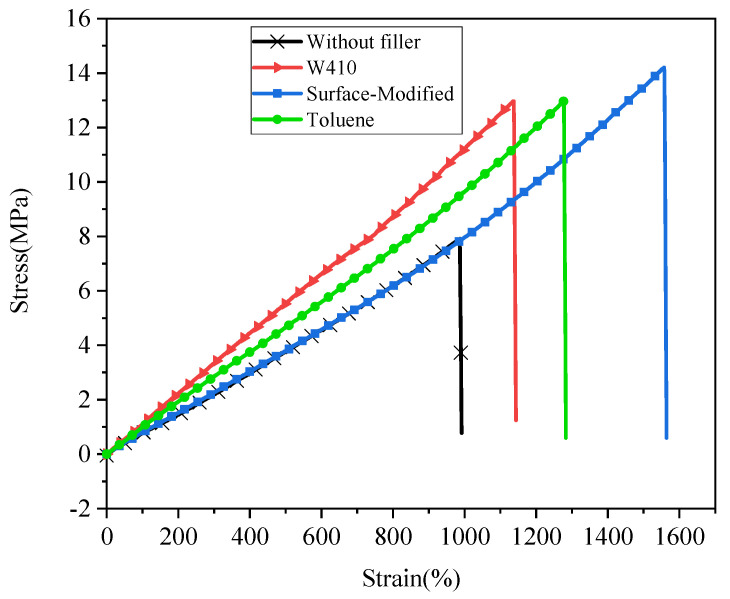
Tensile stress vs. strain for the without-filler compound and nanocomposites.

The tensile stress versus strain (%) of nanocomposites reinforced by nano-CaCO_3_ in comparison to the without-filler compound is graphed in Figure 8. Table 5 presents some mechanical properties obtained from the tensile test of nanocomposites reinforced with calcium carbonate nanoparticles compared to the sample without CaCO3 filler. In this test, properties such as ultimate stress or tensile strength ($\sigma_{u}(\mathrm{Mpa})$), the percentage change of ultimate stress in nanocomposites compared to the sample without reinforcing nanoparticles ($\mathrm{Variation}\;\mathrm{of}\;\sigma_{u}(\%)$), Young’s modulus (E(Mpa)), percentage of changes in Young’s modulus compared to the sample without calcium carbonate nanoparticles (Variation of E(%)), and percentage of elongation at rupture or break ($\varepsilon_{B}(\%)$) were measured. The results, as expected, show an improvement in all the stated mechanical properties in samples with calcium carbonate nanoparticles compared to the sample without. A careful review of these results indicates that the greatest improvement in ultimate stress is observed in the sample prepared by the surface modification method. The tensile strength of the surface-modified sample compared to the control compound (without filler) increases significantly, by 80%; tensile strength increases 64% for W410 and 63.7% for toluene. In addition, the Young’s modulus results in all dispersion methods show a significant increase compared to the unreinforced compound; the largest increase (approximately 42%) is demonstrated with the W410. The sample prepared with toluene solvent shows an increase of 18%, and the nanocomposite prepared by the surface modification method shows an increase in stiffness (Young’s modulus) of about 13.7%. These results are in good agreement with the results of the rheometer and the curing test. As mentioned in the previous section, the maximum torque and the difference between the maximum and minimum torque are used as measures to predict stiffness. The highest percentage increase was observed in the sample prepared with W410, followed by toluene and finally the surface modification method.

The last column of Table 5 also contains data on the percentage of sample elongation at rupture. As a general rule, in most materials, as Young’s modulus increases, the percentage of elongation decreases. Prepared nanocomposite specimens do not follow this rule, and observations show that despite the rise in Young’s modulus, the percentage of elongation increased with the addition of calcium carbonate nanoparticles. This elongation growth can be attributed to the completely spherical shape of CaCO_3_ nanopowders, which, when interacting with the polymer matrix, allows the nanopowders to slide on the chains of the matrix. As a result, instead of decreasing elongation, the interaction between the nanopowders and the matrix causes an increase in elongation. This unique feature, which simultaneously increases strength and increases deformation, can be used in the design and manufacturing of automotive tires.

**Figure 9:**
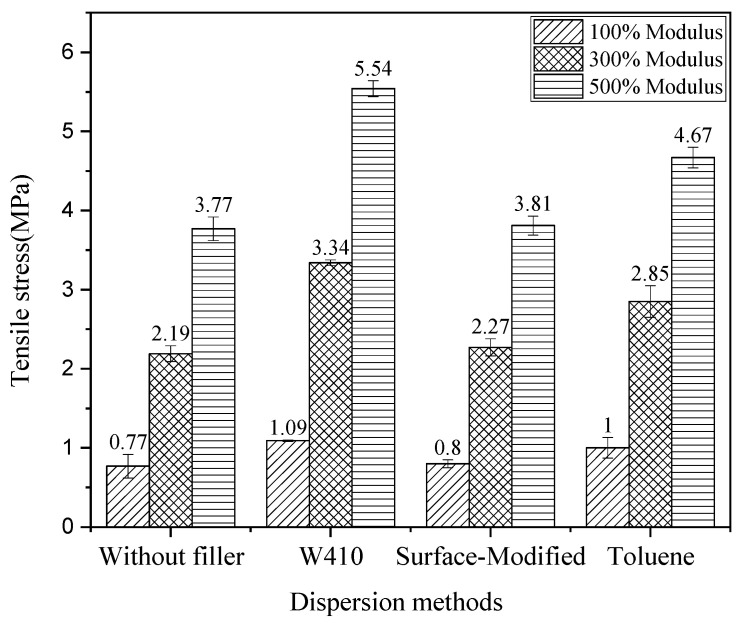
Stress at 100%, 300%, and 500% strain for the without-filler compound and nanocomposites.

Figure 9 shows that the highest tensile strength and 100%, 300%, and 500% elongation, or 100%, 300%, and 500% modulus, were found for the W410-solvent method, followed by the solvent method with toluene; the smallest increase was found in the surface-modified sample.

Results are in good agreement with the outcomes of other research, in which it has been shown that tensile properties of rubber composites with both uncoated calcium carbonates and stearic acid-coated calcium carbonate particles are improved and have similar tensile properties and rolling resistance as silica-reinforced rubber. Furthermore, the results illustrate that in the surface modification method, stearic acid-coated calcium carbonate nanoparticles cause a greater reinforcement effect in rupture and higher ultimate stress than the uncoated calcium carbonate particles [41,42].

### 3.5. Mechanical Properties—Tear Test

**Figure 10:**
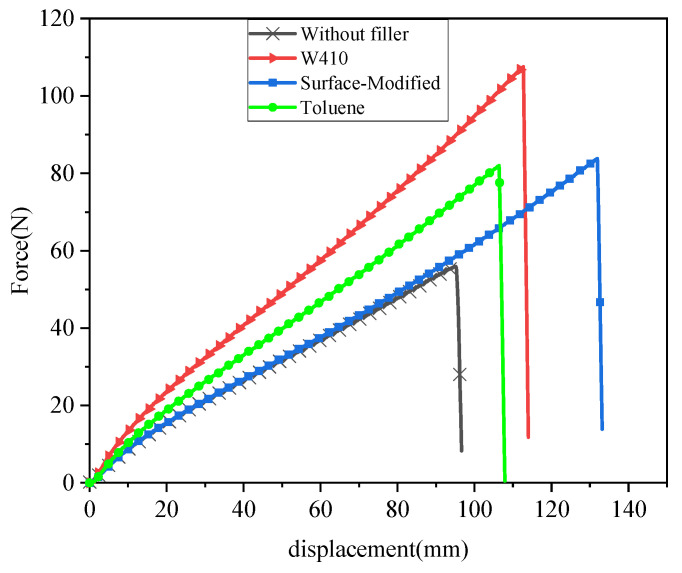
Tear force vs. displacement for the without-filler compound and nanocomposites.

The tear force versus displacement of samples reinforced with nano-CaCO_3_ in comparison to samples without filler compound are plotted in Figure 10. Some of the mechanical properties obtained from the tear test are illustrated in Table 6. In this test, properties such as ultimate tear stress or tear strength, percentage of change in tear strength compared to the sample without reinforcing nano-CaCO_3_ particles, and percentage of elongation to tear were measured.

The highest tear strength is observed in the W410-solvent sample, which increased by 80% compared to the control compound. The highest ultimate tear stress was observed in the surface modification method, with an increase of about 57%. Finally, the solvent method with toluene showed a 50% improvement and increase in tear resistance. The small size of calcium carbonate nanoparticles helps prevent crack propagation, which causes failure and rupture. As a result, the proper distribution of nanoparticles leads to increased tear strength. Researchers have investigated the effect of the addition of macro-, micro-, and nanoscale fillers to natural rubber on tear strength and found that nanofillers can improve tear strength significantly [43].

As seen in tensile tests for all nanocomposites, the percentage of elongation at rupture rises as tensile strength increases. Similarly, the failure strain increases in the tear test as well. The highest percentage increase (268%) is seen with the surface modification method, followed by the W410-solvent method (229%), the toluene sample (215%), and finally the compound without nano-CaCO_3_ (193%).

### 3.6. Mechanical Properties—Hardness Test

Table 7 shows the Shore A hardness number for samples without nanoparticles and samples with calcium carbonate nanoparticles (3.52 wt%) for various dispersion methods. The table illustrates the percentage hardness change compared to the sample without nano-CaCO_3_. The results reveal that, in all methods, adding calcium carbonate nanoparticles increases hardness compared to the sample without nanoparticles; the largest increase is seen in the solvent 410 method (about 17%), followed by the toluene method (about 7.5%), with the smallest increase in hardness seen in the surface modification method (about 3.77%).

The hardness of the material is proportional to its elastic modulus in small deformation or elongation and, as seen in the tensile test results, the highest increase in the modulus of elasticity, or Young’s modulus, is seen in the W410 solvent method and the lowest in the surface-modified method. At the same time, the accuracy of these results can be verified by comparing them with the results of the curing properties because the stronger bonds between nanoparticles and polymer chains cause the maximum torque and the difference between maximum torque and minimum torque, which are measures of the elastic and shear modulus, to increase as well. By knowing the relationship between the hardness number and the stiffness modulus in small deformations and comparing the results with tensile and rheometer tests, the accuracy of the results is verified. Other researchers have analyzed the effect of adding different fillers such as silica, carbon black, and CaCO_3_ to the rubber matrix on mechanical properties. The hardness of natural rubber vulcanisates filled with micro- or nanosized fillers has been studied, and the results illustrate that the hardness of filled-NR vulcanisates increases with filler loading; the percentage of this growth is entirely related to particle size, dispersion method, the weight percent of fillers, and the filler’s characteristics [44,45,46,47].

### 3.7. Mechanical Properties—Resilience Test

The rebound resilience of rubber compounds is the measure of their elasticity and flexibility when exposed to various stresses. Resilience is the ratio of the energy released in the deformation recovery to the energy that caused the deformation. Measuring tire flexibility and elasticity can be useful to choose the right material for a particular application. The following formula is used to calculate rebound resilience.

(5) $$\mathrm{Rebound}\;\mathrm{resiliance}(\%)=\frac{1-\cos\theta_{2}}{1-\cos\theta_{1}}\times 100$$

In the above equation, $\theta_{1}$ and $\theta_{2}$ are the initial angle of the pendulum before release and the rebound angle of the pendulum after impact on the specimen, respectively.

Table 8 shows the rebound resilience for nano-free samples and nanosamples with a weight percentage of 3.52% calcium carbonate nanoparticles for various dispersion methods. The table displays the percentage change in resilience compared to the sample without nanofiller.

The results show that, with the addition of calcium carbonate nanoparticles in all methods, the resilience is reduced compared to the sample without nanoparticles; the highest percentage of reduction is related to the solvent-W410 method (about 10.64%), followed by the toluene method (about 6.38%), with the lowest reduction observed in the surface-modified samples (4.25%). The rebound resilience property is proportional to the elasticity of the specimens and their frictional resistance. The addition of calcium carbonate nanoparticles, as observed in the tensile test, increases the modulus of elasticity, so this reduction in resilience is in good agreement with the tensile test results. The resilience reduction in nanocomposites can be attributed to the greater energy loss at the interface between the matrix and the nanoparticles. The stronger bonds between the nanoparticles and the polymer cause the greater reduction in rebound resilience. As shown in the results of the curing properties, the resilience test results verify the rheometer test results as well. Studies have shown that a decrease in rebound resilience can lead to an increase in mechanical properties such as hardness, stiffness, modulus, abrasion resistance, tear resistance, and the frictional coefficient for traction [48,49].

## 4. Conclusions

Fillers are added to rubbers for various reasons, including mechanical reinforcement, modification of electrical or thermal conductivity, and ease of processing. The properties and processability of the composite can be affected by various factors, including filler size and geometry, dispersion quality, and filler volume fraction. This study focuses on the preparation of nanocomposites based on natural rubber and styrene-butadiene for car tire production. The nanocomposites include various materials such as stearic acid, paraffin wax, and carbon black. The effect of dispersion quality was investigated using different mixing methods and surface modifications.

X-ray diffraction and SEM were used to analyze the nanocomposites. Nanoparticle size was found to be around 34 nm, with increased interlayer space and diffraction peaks. This indicates good dispersion of calcium carbonate nanoparticles in rubber. SEM images show nanoparticle size in the range of 10–60 nm, consistent with XRD results and calcium carbonate nanoparticle specifications.

The curing characteristics of nanocomposites were analyzed by measuring various properties, including scorch time, cure times, torque, peak cure rate, and reinforcement efficiency. Results indicate that adding nanoparticles results in faster curing, with the solvent-W410 method producing the fastest results. Maximum torque and the difference between minimum and maximum torque were also increased by the addition of calcium carbonate nanoparticles.

To study the improvement of mechanical properties, tensile, tear, hardness, and resilience tests for composite samples without nanofillers and nanocomposites were performed. The mechanical properties obtained from the tensile test of nanocomposites illustrate the improvement of tensile properties in all methods compared to the sample without CaCO_3_. The greatest improvement in ultimate stress was seen in the surface modification method (80%), followed by the W410 method (64%), and the toluene method (63.7%). The results demonstrate that stearic acid-coated nano-CaCO_3_ in the surface modification method has a greater reinforcement effect on rupture strain and maximum ultimate stress than the other dispersion methods. Furthermore, the results show that the elastic properties of nanocomposites increase, but in contrast to the maximum ultimate stress and elongation at break, which belong to the surface modification sample, the highest Young’s modulus is observed in the W410-solvent method. In the tear test, the highest tear strength is observed in the W410-solvent sample (80%), followed by the surface modification method (57%), and finally the solvent-toluene method, which shows a 50% improvement compared to the non-filler composition. Shore A hardness numbers measured by the hardness test indicate that the hardest sample is W410, followed by the toluene sample; the lowest hardness corresponds to the surface-modified samples. The results of the resilience test show that with the addition of CaCO_3_ nanofillers in all dispersion methods, rebound resilience is reduced compared to the sample without filler. The highest percentage drop is related to W410 (approximately 10.64%), followed by toluene (approximately 6.38%); the lowest reduction is observed in surface-modified samples (about 4.25%). As the rebound resilience properties are related to the elastic properties of the specimens, the addition of calcium carbonate nanoparticles increases the Young’s modulus, as observed in the tensile test properties as well; thus, decreased resilience verifies the tensile and rheometer test results.

## Figures and Tables

**Table 1:** The compositions of the nanocomposites.

Material	Phr (Parts per Hundred Rubber)	Weight (g)	Weight Percent (wt%)
NR	50.02	367.00	29.36
SBR	49.98	366.70	29.34
ST.AC	3.45	25.31	2.03
Nano Caco3	6	44.02	3.52
Pwax	1.78	13.06	1.04
AR OIL	5.22	38.30	3.06
CB N500	39.1	286.88	22.95
ZnO	5.96	43.73	3.50
CBS	0.76	5.58	0.45
Sulfur	2.82	20.69	1.66
6PPD	2.64	19.37	1.55
TMQ	2.64	19.37	1.55
Total	170.37	1250	100

**Table 2:** Characteristics of the nano-CaCO_3_ used in this research.

Appearance	White powder
Morphology	Cubic–hexagonal
Molecular Weight	100.09
Density	2.93 g/cm^3^
Mean particular diameter	10–60 nm

**Table 3:** The amount of solvent added for the preparation of nanocomposites (solvent 410 and solvent toluene).

Solute	Solvent (mL)	
	Solvent W410	Solvent TOLUENE
Natural Rubber (NR)	2000	1200
Styrene-Butadiene Rubber (SBR)	750	700

**Table 4:** Cure rheometry properties for different dispersion methods.

Nano-Composites	M_L_ (*lbf .in*)	M_H_ (*lbf .in*)	ΔM (*lbf .in*)	ts_5_(min)	ts_10_(min)	tc_90_(min)	tc_95_(min)	$V_{\max}(\frac{\%}{\min})$	CRI(min^−1^)
Without filler	15.13	72.6	57.47	1.55	1.77	6.4	7.57	25.48	16.61
W410	16.84	96.2	79.36	1.23	1.41	5.2	6.12	30.54	20.45
Surface-Modified	15.38	79.8	64.42	1.57	1.78	6	6.88	26.47	18.83
Toluene	19.73	86.1	66.37	1.28	1.47	5.8	6.73	26.25	18.34

**Table 5:** Mechanical properties of tensile test for different dispersion methods.

Nanocomposites	σ_u_(MPa)	Variation of σ_u_ (%)	E(MPa)	Variation of E(%)	ε_B_(%)
Without filler	7.91	-	0.8	-	986.5
W410	12.98	64%	1.14	42%	1139.1
Surfaced-Modified	14.21	80%	0.91	13.7%	1557.5
Toluene	12.95	63.7%	0.95	18.1%	1367.4

**Table 6:** Mechanical properties of tear test for different dispersion methods.

Nanocomposites	Tear Strength	Variation of Tear Strenght(%)	ε_B_−Tear(%)
Without filler	1.41	-	193
W410	2.54	80%	229
Surfaced-Modified	2.22	57%	268
Toluene	2.12	50.5%	215

**Table 7:** Mechanical properties of the hardness test for different dispersion methods.

Nanocomposites	Hardness (Shroe A)	Variation of Hardness(%)
Without filler	53	-
W410	62	16.98
Surfaced-Modified	55	3.77
Toluene	57	7.54

**Table 8:** Mechanical properties of the rebound resilience test for different dispersion methods.

Nanocomposites	Rebound Resilience (%)	Variation of Rebound Resilience (%)
Without filler	47	-
W410	42	−10.64
Surfaced-Modified	45	−4.25
Toluene	44	−6.38

## Data Availability

Data will be available upon request.
